# 2-(Morpholinium-4-yl)ethyl­ammonium sulfate methanol monosolvate

**DOI:** 10.1107/S1600536810010846

**Published:** 2010-03-27

**Authors:** Ye Bi

**Affiliations:** a College of Chemistry and Chemical Engineering, Qiqihar University, Qiqihar 161006, People’s Republic of China

## Abstract

In the title compound, C_6_H_16_N_2_O^2+^·SO_4_^2−^·CH_3_OH, the morpholinium ring of the dication adopts a chair conformation. The crystal structure is stabilized by an extensive three-dimensional network of inter­molecular O—H⋯O, N—H⋯O, O—H⋯S and N—H⋯S hydrogen bonds.

## Related literature

For supra­molecular compounds derived from the self-assembly of inorganic acids with organic amines, see: Xu (2010 ▶); Akhtar *et al.* (2010 ▶); Zhang & Liu (2010 ▶); Hemamalini & Fun (2010 ▶); SiMa (2010 ▶).
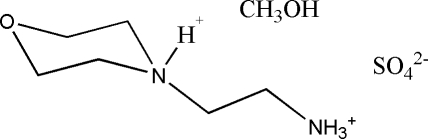


## Experimental

### 

#### Crystal data

C_6_H_16_N_2_O^2+^·SO_4_^2−^·CH_4_O
*M* *_r_* = 260.31
Monoclinic, 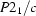

*a* = 15.593 (14) Å
*b* = 8.573 (8) Å
*c* = 9.483 (9) Å
β = 106.395 (11)°
*V* = 1216.0 (19) Å^3^
*Z* = 4
Mo *K*α radiation
μ = 0.28 mm^−1^
*T* = 298 K
0.20 × 0.18 × 0.18 mm

#### Data collection

Bruker SMART 1000 CCD area-detector diffractometer
Absorption correction: multi-scan (*SADABS*; Sheldrick, 1996 ▶) *T*_min_ = 0.946, *T*_max_ = 0.951
5674 measured reflections
2462 independent reflections
1726 reflections with *I* > 2σ(*I*)
*R*_int_ = 0.049

#### Refinement

*R*[*F* ^2^ > 2σ(*F* ^2^)] = 0.087
*wR*(*F* ^2^) = 0.286
*S* = 1.08
2462 reflections
151 parameters
1 restraint
H atoms treated by a mixture of independent and constrained refinement
Δρ_max_ = 0.62 e Å^−3^
Δρ_min_ = −0.87 e Å^−3^

### 

Data collection: *SMART* (Bruker, 1998 ▶); cell refinement: *SAINT* (Bruker, 1998 ▶); data reduction: *SAINT*; program(s) used to solve structure: *SHELXS97* (Sheldrick, 2008 ▶); program(s) used to refine structure: *SHELXL97* (Sheldrick, 2008 ▶); molecular graphics: *SHELXTL* (Sheldrick, 2008 ▶); software used to prepare material for publication: *SHELXTL*.

## Supplementary Material

Crystal structure: contains datablocks global, I. DOI: 10.1107/S1600536810010846/sj2757sup1.cif

Structure factors: contains datablocks I. DOI: 10.1107/S1600536810010846/sj2757Isup2.hkl

Additional supplementary materials:  crystallographic information; 3D view; checkCIF report

## Figures and Tables

**Table 1:** Hydrogen-bond geometry (Å, °)

*D*—H⋯*A*	*D*—H	H⋯*A*	*D*⋯*A*	*D*—H⋯*A*
O5—H5⋯O4^i^	0.82	1.85	2.659 (6)	172
O5—H5⋯S1^i^	0.82	2.92	3.636 (6)	147
N2—H2*A*⋯O1^ii^	0.89	2.07	2.914 (5)	158
N2—H2*A*⋯O3^ii^	0.89	2.30	3.001 (5)	135
N2—H2*A*⋯S1^ii^	0.89	2.68	3.553 (4)	167
N2—H2*B*⋯O2^iii^	0.89	2.02	2.898 (5)	168
N2—H2*B*⋯O3^iii^	0.89	2.45	3.081 (5)	128
N2—H2*B*⋯S1^iii^	0.89	2.72	3.567 (4)	160
N2—H2*C*⋯O2	0.89	2.18	2.997 (5)	153
N2—H2*C*⋯O1	0.89	2.23	2.930 (4)	135
N2—H2*C*⋯S1	0.89	2.71	3.565 (4)	162
N1—H1⋯O2^iv^	0.90 (1)	2.01 (3)	2.837 (5)	152 (5)
N1—H1⋯O4^iv^	0.90 (1)	2.60 (3)	3.382 (6)	146 (5)
N1—H1⋯S1^iv^	0.90 (1)	2.85 (1)	3.738 (4)	172 (5)

Symmetry codes: (i) 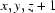; (ii) 
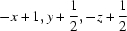
; (iii) 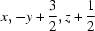; (iv) 
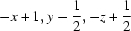
.

**Fig. 1.**
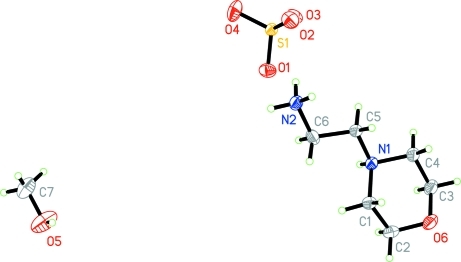
The structure of the title compound, showing 30% probability displacement ellipsoids and the atom-numbering scheme.

**Fig. 2.**
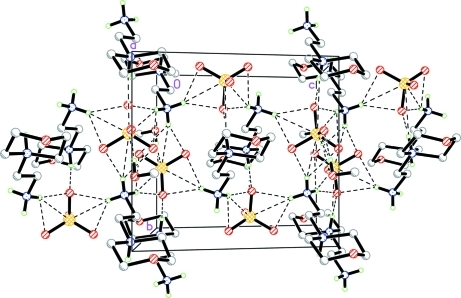
Molecular packing of the title compound, viewed along the a axis. Hydrogen bonds are shown as dashed lines.

## supplementary crystallographic information

### Comment

Self-assembly of inorganic acids with organic amines readily gives rise to hydrogen-bonded supramolecular compounds (Xu, 2010; Akhtar *et al.*, 2010; Zhang & Liu, 2010; Hemamalini & Fun, 2010; SiMa, 2010). In order to construct a similar supramolecular compound, the title compound was prepared from the reaction of 2-morpholin-4-ylethylamine with sulfuric acid in a methanol solution and its structure is reported here.

The title compound consists of a 2-morpholin-4-ylethylammonium dication, a sulfate dianion, and a methanol molecule (Fig. 1). The crystal structure is stabilized by intermolecular O–H···O, N–H···O, O–H···S, and N–H···S hydrogen bonds (Table 1, Fig. 2).

### Experimental

Equimolar quantities (1.0 mmol each) of 2-morpholin-4-ylethylamine and sulfuric acid were mixed in a methanol solution. The mixture was stirred at room temperature for half an hour to give a colorless solution. After keeping the solution in air for a few days, colorless block-shaped crystals were formed.

### Refinement

H1 attached to N1 was located from a difference map and refined isotropically, with the N–H distance restrained to 0.90 (1) Å. Other H atoms were placed in calculated positions and constrained to ride on their parent atoms with C–H distances of 0.96-0.97 Å, N–H distances of 0.89 Å, O–H distance of 0.82 Å, and with U_iso_(H) set to 1.2U_eq_(C,*N*) and 1.5U_eq_(O5 and C7). Crystals were small and very weakly diffracting and this is reflected in the low fraction of measured reflections and the relatively poor residuals.

### Crystal data

**Table:**

C_6_H_16_N_2_O^2+^·SO_4_^2^^−^·CH_4_O	*F*(000) = 560
*M**_r_* = 260.31	*D*_x_ = 1.422 Mg m^−^^3^
Monoclinic, *P*2_1_/*c*	Mo *K*α radiation, λ = 0.71073 Å
Hall symbol: -P 2ybc	Cell parameters from 2104 reflections
*a* = 15.593 (14) Å	θ = 2.2–27.7°
*b* = 8.573 (8) Å	µ = 0.28 mm^−^^1^
*c* = 9.483 (9) Å	*T* = 298 K
β = 106.395 (11)°	Block, colorless
*V* = 1216.0 (19) Å^3^	0.20 × 0.18 × 0.18 mm
*Z* = 4	

### Data collection

**Table:**

Bruker SMART 1000 CCD area-detector diffractometer	2462 independent reflections
Radiation source: fine-focus sealed tube	1726 reflections with *I* > 2σ(*I*)
graphite	*R*_int_ = 0.049
ω scans	θ_max_ = 27.0°, θ_min_ = 2.7°
Absorption correction: multi-scan (*SADABS*; Sheldrick, 1996)	*h* = −19→14
*T*_min_ = 0.946, *T*_max_ = 0.951	*k* = −10→10
5674 measured reflections	*l* = −10→12

### Refinement

**Table:**

Refinement on *F*^2^	Primary atom site location: structure-invariant direct methods
Least-squares matrix: full	Secondary atom site location: difference Fourier map
*R*[*F*^2^ > 2σ(*F*^2^)] = 0.087	Hydrogen site location: inferred from neighbouring sites
*wR*(*F*^2^) = 0.286	H atoms treated by a mixture of independent and constrained refinement
*S* = 1.08	*w* = 1/[σ^2^(*F*_o_^2^) + (0.1965*P*)^2^] where *P* = (*F*_o_^2^ + 2*F*_c_^2^)/3
2462 reflections	(Δ/σ)_max_ < 0.001
151 parameters	Δρ_max_ = 0.62 e Å^−^^3^
1 restraint	Δρ_min_ = −0.87 e Å^−^^3^

### Special details

**Table:**

Geometry. All esds (except the esd in the dihedral angle between two l.s. planes) are estimated using the full covariance matrix. The cell esds are taken into account individually in the estimation of esds in distances, angles and torsion angles; correlations between esds in cell parameters are only used when they are defined by crystal symmetry. An approximate (isotropic) treatment of cell esds is used for estimating esds involving l.s. planes.
Refinement. Refinement of *F*^2^ against ALL reflections. The weighted *R*-factor wR and goodness of fit *S* are based on *F*^2^, conventional *R*-factors *R* are based on *F*, with *F* set to zero for negative *F*^2^. The threshold expression of *F*^2^ > σ(*F*^2^) is used only for calculating *R*-factors(gt) etc. and is not relevant to the choice of reflections for refinement. *R*-factors based on *F*^2^ are statistically about twice as large as those based on *F*, and *R*- factors based on ALL data will be even larger.

### Fractional atomic coordinates and isotropic or equivalent isotropic displacement parameters (Å^2^)

**Table:**

	*x*	*y*	*z*	*U*_iso_*/*U*_eq_	
S1	0.35803 (6)	0.59946 (11)	0.06772 (9)	0.0369 (4)	
O1	0.3987 (2)	0.5157 (3)	0.2047 (3)	0.0523 (8)	
O2	0.3969 (2)	0.7579 (3)	0.0786 (3)	0.0557 (8)	
O3	0.3768 (2)	0.5166 (4)	−0.0531 (3)	0.0574 (9)	
O4	0.2617 (2)	0.6167 (5)	0.0429 (5)	0.0772 (11)	
O5	0.1552 (3)	0.3688 (5)	1.0088 (7)	0.1071 (18)	
H5	0.1834	0.4507	1.0182	0.161*	
O6	0.9107 (2)	0.4451 (4)	0.5902 (3)	0.0606 (9)	
N1	0.7246 (2)	0.5104 (4)	0.4511 (3)	0.0371 (7)	
N2	0.51382 (19)	0.7510 (4)	0.3894 (3)	0.0403 (8)	
H2A	0.5402	0.8420	0.3841	0.061*	
H2B	0.4754	0.7621	0.4426	0.061*	
H2C	0.4846	0.7192	0.2993	0.061*	
C1	0.7681 (3)	0.5356 (5)	0.6131 (4)	0.0469 (10)	
H1A	0.7247	0.5183	0.6673	0.056*	
H1B	0.7893	0.6422	0.6300	0.056*	
C2	0.8462 (3)	0.4232 (7)	0.6667 (5)	0.0593 (12)	
H2D	0.8736	0.4392	0.7709	0.071*	
H2E	0.8242	0.3168	0.6528	0.071*	
C3	0.8723 (3)	0.4061 (6)	0.4418 (5)	0.0558 (12)	
H3A	0.8513	0.2991	0.4351	0.067*	
H3B	0.9174	0.4135	0.3895	0.067*	
C4	0.7949 (3)	0.5129 (5)	0.3698 (4)	0.0471 (10)	
H4A	0.8168	0.6186	0.3682	0.056*	
H4B	0.7684	0.4801	0.2690	0.056*	
C5	0.6558 (2)	0.6314 (5)	0.3848 (4)	0.0394 (8)	
H5A	0.6304	0.6094	0.2810	0.047*	
H5B	0.6841	0.7330	0.3938	0.047*	
C6	0.5817 (3)	0.6351 (5)	0.4589 (5)	0.0497 (10)	
H6A	0.5542	0.5328	0.4524	0.060*	
H6B	0.6065	0.6604	0.5621	0.060*	
C7	0.0750 (4)	0.3876 (9)	0.9066 (8)	0.0911 (19)	
H7A	0.0295	0.3351	0.9386	0.137*	
H7B	0.0778	0.3444	0.8146	0.137*	
H7C	0.0611	0.4968	0.8944	0.137*	
H1	0.702 (4)	0.413 (3)	0.437 (6)	0.080*	

### Atomic displacement parameters (Å^2^)

**Table:**

	*U*^11^	*U*^22^	*U*^33^	*U*^12^	*U*^13^	*U*^23^
S1	0.0318 (6)	0.0445 (6)	0.0335 (6)	0.0004 (3)	0.0079 (4)	0.0002 (3)
O1	0.0618 (19)	0.0560 (18)	0.0355 (14)	−0.0019 (14)	0.0078 (13)	0.0054 (11)
O2	0.068 (2)	0.0439 (17)	0.0592 (18)	−0.0076 (14)	0.0246 (14)	−0.0018 (12)
O3	0.071 (2)	0.065 (2)	0.0364 (14)	0.0049 (15)	0.0159 (13)	−0.0076 (12)
O4	0.0357 (18)	0.089 (3)	0.107 (3)	0.0122 (15)	0.0190 (18)	0.005 (2)
O5	0.070 (3)	0.081 (3)	0.143 (4)	−0.002 (2)	−0.016 (3)	0.016 (3)
O6	0.0330 (15)	0.092 (2)	0.0519 (17)	0.0065 (15)	0.0046 (12)	−0.0012 (16)
N1	0.0305 (15)	0.0446 (17)	0.0346 (15)	−0.0036 (12)	0.0068 (11)	−0.0012 (12)
N2	0.0317 (16)	0.0509 (19)	0.0376 (15)	−0.0020 (12)	0.0085 (12)	−0.0046 (13)
C1	0.039 (2)	0.067 (3)	0.0326 (18)	0.0013 (18)	0.0064 (14)	0.0002 (16)
C2	0.043 (2)	0.089 (3)	0.039 (2)	0.009 (2)	0.0008 (17)	0.011 (2)
C3	0.036 (2)	0.081 (3)	0.050 (2)	0.0041 (19)	0.0122 (17)	−0.0046 (19)
C4	0.036 (2)	0.068 (3)	0.0380 (18)	−0.0050 (18)	0.0119 (15)	−0.0039 (16)
C5	0.0333 (18)	0.055 (2)	0.0287 (16)	0.0000 (15)	0.0072 (13)	−0.0001 (14)
C6	0.050 (2)	0.059 (2)	0.047 (2)	0.0108 (19)	0.0257 (17)	0.0136 (18)
C7	0.049 (3)	0.108 (5)	0.109 (5)	0.005 (3)	0.011 (3)	0.000 (4)

### Geometric parameters (Å, °)

**Table:**

S1—O3	1.446 (3)	C1—H1A	0.9700
S1—O4	1.461 (4)	C1—H1B	0.9700
S1—O1	1.463 (3)	C2—H2D	0.9700
S1—O2	1.479 (3)	C2—H2E	0.9700
O5—C7	1.358 (7)	C3—C4	1.516 (6)
O5—H5	0.8200	C3—H3A	0.9700
O6—C3	1.405 (5)	C3—H3B	0.9700
O6—C2	1.409 (6)	C4—H4A	0.9700
N1—C5	1.497 (5)	C4—H4B	0.9700
N1—C4	1.508 (5)	C5—C6	1.512 (5)
N1—C1	1.509 (5)	C5—H5A	0.9700
N1—H1	0.899 (10)	C5—H5B	0.9700
N2—C6	1.466 (5)	C6—H6A	0.9700
N2—H2A	0.8900	C6—H6B	0.9700
N2—H2B	0.8900	C7—H7A	0.9600
N2—H2C	0.8900	C7—H7B	0.9600
C1—C2	1.524 (6)	C7—H7C	0.9600
			
O3—S1—O4	110.5 (2)	H2D—C2—H2E	108.0
O3—S1—O1	109.1 (2)	O6—C3—C4	111.5 (4)
O4—S1—O1	111.2 (2)	O6—C3—H3A	109.3
O3—S1—O2	109.6 (2)	C4—C3—H3A	109.3
O4—S1—O2	107.5 (2)	O6—C3—H3B	109.3
O1—S1—O2	108.85 (17)	C4—C3—H3B	109.3
C7—O5—H5	109.5	H3A—C3—H3B	108.0
C3—O6—C2	108.6 (3)	N1—C4—C3	111.3 (3)
C5—N1—C4	108.3 (3)	N1—C4—H4A	109.4
C5—N1—C1	113.0 (3)	C3—C4—H4A	109.4
C4—N1—C1	109.6 (3)	N1—C4—H4B	109.4
C5—N1—H1	112 (4)	C3—C4—H4B	109.4
C4—N1—H1	104 (4)	H4A—C4—H4B	108.0
C1—N1—H1	109 (4)	N1—C5—C6	111.7 (3)
C6—N2—H2A	109.5	N1—C5—H5A	109.3
C6—N2—H2B	109.5	C6—C5—H5A	109.3
H2A—N2—H2B	109.5	N1—C5—H5B	109.3
C6—N2—H2C	109.5	C6—C5—H5B	109.3
H2A—N2—H2C	109.5	H5A—C5—H5B	107.9
H2B—N2—H2C	109.5	N2—C6—C5	110.8 (3)
N1—C1—C2	109.6 (3)	N2—C6—H6A	109.5
N1—C1—H1A	109.7	C5—C6—H6A	109.5
C2—C1—H1A	109.7	N2—C6—H6B	109.5
N1—C1—H1B	109.7	C5—C6—H6B	109.5
C2—C1—H1B	109.7	H6A—C6—H6B	108.1
H1A—C1—H1B	108.2	O5—C7—H7A	109.5
O6—C2—C1	111.3 (4)	O5—C7—H7B	109.5
O6—C2—H2D	109.4	H7A—C7—H7B	109.5
C1—C2—H2D	109.4	O5—C7—H7C	109.5
O6—C2—H2E	109.4	H7A—C7—H7C	109.5
C1—C2—H2E	109.4	H7B—C7—H7C	109.5

### Hydrogen-bond geometry (Å, °)

**Table:**

*D*—H···*A*	*D*—H	H···*A*	*D*···*A*	*D*—H···*A*
O5—H5···O4^i^	0.82	1.85	2.659 (6)	172
O5—H5···S1^i^	0.82	2.92	3.636 (6)	147
N2—H2A···O1^ii^	0.89	2.07	2.914 (5)	158
N2—H2A···O3^ii^	0.89	2.30	3.001 (5)	135
N2—H2A···S1^ii^	0.89	2.68	3.553 (4)	167
N2—H2B···O2^iii^	0.89	2.02	2.898 (5)	168
N2—H2B···O3^iii^	0.89	2.45	3.081 (5)	128
N2—H2B···S1^iii^	0.89	2.72	3.567 (4)	160
N2—H2C···O2	0.89	2.18	2.997 (5)	153
N2—H2C···O1	0.89	2.23	2.930 (4)	135
N2—H2C···S1	0.89	2.71	3.565 (4)	162
N1—H1···O2^iv^	0.90 (1)	2.01 (3)	2.837 (5)	152 (5)
N1—H1···O4^iv^	0.90 (1)	2.60 (3)	3.382 (6)	146 (5)
N1—H1···S1^iv^	0.90 (1)	2.85 (1)	3.738 (4)	172 (5)

Symmetry codes: (i) *x*, *y*, *z*+1; (ii) −*x*+1, *y*+1/2, −*z*+1/2; (iii) *x*, −*y*+3/2, *z*+1/2; (iv) −*x*+1, *y*−1/2, −*z*+1/2.
